# Editorial: Insights and innovations in consumer neuroscience

**DOI:** 10.3389/fnhum.2026.1924520

**Published:** 2026-07-20

**Authors:** Riccardo Valesi, Margherita Zito, Chiara Casiraghi, Vincenzo Russo

**Affiliations:** 1 Department of Management, Università degli Studi di Bergamo, Bergamo, Italy; 2 Neuro Lab for Business and Society – Neuroscience Laboratory, Università degli Studi di Bergamo, Bergamo, Italy; 3 Department of Business, Law, Economics and Consumer Behaviour “Carlo A. Ricciardi”, Università IULM, Milan, Italy; 4 Behavior and Brain Lab IULM – Neuromarketing Research Centre, Università IULM, Milan, Italy

**Keywords:** consumer behavior, consumer neuroscience, decision making, implicit measures, neuromarketing, neuroscience methods, psychophysiology

In an era marked by rapid social, economic, and technological advancement, which has significantly shaped the marketing landscape, neuroscience has emerged as a set of methods capable of generating valuable insights into marketing, consumer behavior, and advertising. Conceptualized as the application of neuroscientific technologies to the marketing sector (Lee et al., 2007), consumer neuroscience has rapidly gained traction, contributing to multiple areas of inquiry, including the role of emotions in decision-making, socially driven mechanisms, and perceptual influences on behavior (Casado-Aranda et al., 2023). In business, its application has come to span a broad range of sectors, covering fashion, fast-moving consumer goods, and the automotive industry (Oliveira et al., 2022), while also informing key marketing practices such as influencer marketing, brand development, and advertising (Cenizo, 2025). Although economic and expertise-related barriers initially confined the adoption of these techniques predominantly to leading companies, technological simplification has progressively facilitated their diffusion beyond such firms (Bolls et al., 2025), increasingly extending their use to small businesses seeking to enhance economic performance (Malodia et al., 2022). This expansion is driven by the distinctive advantages of neuroscientific approaches, which can uncover automatic psychological reactions underlying consumer behavior (Friedmann et al., 2024), mitigate biases inherent in self-reported measures, and enable the continuous assessment of consumers' responses to specific messages (Casado-Aranda et al., 2022).

Notwithstanding the potential of these techniques, research into their application remains limited and fragmented, and their use also poses ethical challenges concerning consumer autonomy and privacy. This Research Topic presents four manuscripts that offer an up-to-date overview of consumer neuroscience and psychological approaches, highlighting their potential to inform marketing strategies and their current application across contemporary research contexts.

Eremenko et al. open the Research Topic by examining whether multisensory retail environments elicit measurable emotional responses and support emotion-based retail zoning. Drawing on sensory marketing and the S–O–R model, the study first maps visual, auditory, and olfactory stimuli onto target zone emotions in 46 participants, then tests nine multisensory virtual-reality rooms in 27 participants using heart rate (HR), heart rate variability (HRV), and electrodermal activity (EDA). Findings show coherent cross-modal associations and refined spatial differentiation across experiential zones. HRV increases with perceived pleasantness, whereas HR and EDA show no significant associations. The study supports biometric pretesting and data-driven store atmospherics in retail design.

Idesis et al. review cognitive state monitoring in neuroadaptive information visualization and its potential to address information overload. Through an analysis of non-invasive tools, including EEG, fNIRS, HRV, EDA, and eye-tracking, the authors map neurophysiological signatures of mental fatigue, cognitive workload, stress, and mind wandering, showing how these states affect users' ability to extract meaning from visual data. They also discuss the role of machine learning in enabling adaptive responses when users become overloaded, distracted, or disengaged, highlighting a promising frontier for research and practice.

Xilai et al. investigate how impulse buying emerges in algorithmically mediated purchasing environments. Drawing on Dual-Process Theory and the S–O–R framework, they develop a dual-route model tested on 564 Chinese consumers assigned to livestream vs. conventional marketplace contexts. Findings show that livestream settings, shaped by social immersion and time pressure, trigger impulsive action through fast heuristic processing, whereas conventional marketplaces operate through a slower route that indirectly reinforces decision confidence. Methodologically, the study combines PLS-SEM to capture linear and mediating effects with fsQCA to identify alternative causal configurations across digital shopping contexts.

Jang et al. offer a complementary perspective by examining the drivers of OTT platform adoption for sports content consumption. Applying an Extended Technology Acceptance Model to 303 Korean viewers, they find that perceived usefulness does not significantly predict usage intention. Instead, adoption is driven by perceived ease of use, enjoyment, and interactivity, suggesting that emotional satisfaction and experiential quality outweigh informational value in sports content consumption. The study thus shows that viewers' attachment to OTT platforms is shaped by deeper emotional and experiential processes, often operating below conscious awareness and central to consumer neuroscience.

This Research Topic offers an added value for research and applications in consumer neuroscience. Indeed, the contributions show that consumption experiences are shaped by dynamic cognitive, affective, and physiological processes. Treating these internal states as core explanatory mechanisms is essential for advancing consumer behavior research and for designing empirically grounded, effective consumer experiences.
